# Immunodiagnosis and Immunotherapeutics Based on Human Papillomavirus for HPV-Induced Cancers

**DOI:** 10.3389/fimmu.2020.586796

**Published:** 2021-01-08

**Authors:** Zhen Dong, Renjian Hu, Yan Du, Li Tan, Lin Li, Juan Du, Longchang Bai, Yingkang Ma, Hongjuan Cui

**Affiliations:** ^1^ State Key Laboratory of Silkworm Genome Biology, Institute of Sericulture and Systems Biology, College of Sericulture & Textile & Biomass Science, Southwest University, Chongqing, China; ^2^ Cancer Center, Reproductive Medicine Center, Medical Research Institute, Southwest University, Chongqing, China; ^3^ NHC Key Laboratory of Birth Defects and Reproductive Health (Chongqing Key Laboratory of Birth Defects and Reproductive Health, Chongqing Population and Family Planning Science and Technology Research Institute), Chongqing, China; ^4^ School of Pharmacy and Bioengineering, Chongqing University of Technology, Chongqing, China; ^5^ Department of Ultrasound, Chongqing University Central Hospital (Chongqing Emergency Medical Center), Chongqing, China; ^6^ Department of Immunology, School of Basic Medicine, Southwest Medical University, Luzhou, China; ^7^ Department of Dermatology, The Third Hospital of Hebei Medical University, Shijiazhuang, China

**Keywords:** cervical cancer, human papillomavirus, monoclonal antibody, immunodiagnosis, immunotherapeutics

## Abstract

Infection with human papillomavirus (HPV) is one of the main causes of malignant neoplasms, especially cervical, anogenital, and oropharyngeal cancers. Although we have developed preventive vaccines that can protect from HPV infection, there are still many new cases of HPV-related cancers worldwide. Early diagnosis and therapy are therefore important for the treatment of these diseases. As HPVs are the major contributors to these cancers, it is reasonable to develop reagents, kits, or devices to detect and eliminate HPVs for early diagnosis and therapeutics. Immunological methods are precise strategies that are promising for the accurate detection and blockade of HPVs. During the last decades, the mechanism of how HPVs induce neoplasms has been extensively elucidated, and several oncogenic HPV early proteins, including E5, E6, and E7, have been shown to be positively related to the oncogenesis and malignancy of HPV-induced cancers. These oncoproteins are promising biomarkers for diagnosis and as targets for the therapeutics of HPV-related cancers. Importantly, many specific monoclonal antibodies (mAbs), or newly designed antibody mimics, as well as new immunological kits, devices, and reagents have been developed for both the immunodiagnosis and immunotherapeutics of HPV-induced cancers. In the current review, we summarize the research progress in the immunodiagnosis and immunotherapeutics based on HPV for HPV-induced cancers. In particular, we depict the most promising serological methods for the detection of HPV infection and several therapeutical immunotherapeutics based on HPV, using immunological tools, including native mAbs, radio-labelled mAbs, affitoxins (affibody-linked toxins), intracellular single-chain antibodies (scFvs), nanobodies, therapeutical vaccines, and T-cell-based therapies. Our review aims to provide new clues for researchers to develop novel strategies and methods for the diagnosis and treatment of HPV-induced tumors.

## Introduction

**Figure 1:**
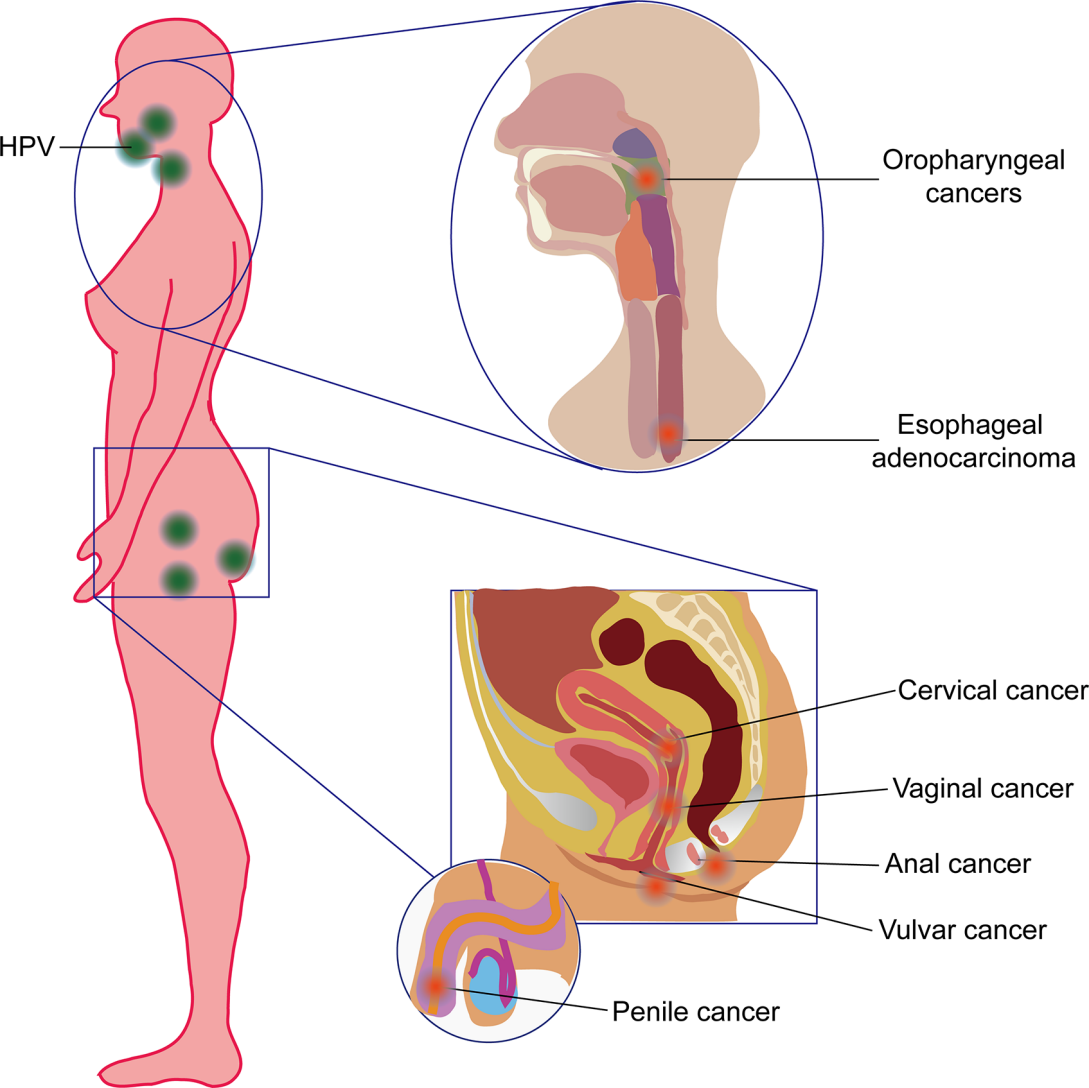
Human papillomavirus (HPV)-induced cancers in human beings. HPV commonly induced oropharyngeal cancer and esophageal adenocarcinoma (also referred to as head and neck cancer) in the orpharynx and esophagus and cervical cancer, vaginal cancer, vulvar cancer, anal cancer, and penile cancer in the reproductive system.

Every year, more than 4.5% (8.6% in women and 0.8% in men) of all cancers worldwide (630,000 new cancer cases), such as cervical cancer, vulvar cancer, vaginal cancer, penile cancer and anal cancer 1), oropharyngeal cancers (OPC; including tumors derived from the base of the tongue and tonsils), and even esophageal adenocarcinoma (EAC) (2, 3), are attributed to the human papillomaviruses (HPVs) (**Figure 1**). Recently, HPV has also been found to be present and active in lung cancer 4) and may also contribute to skin cancers (5). HPVs are a kind of small, unenveloped, and highly host-specific double-stranded circular DNA viruses (6). They are not only microorganisms that are sexually transmitted *via* genital contact but also a kind of viruses that can be passed on by skin and mouth (7). HPVs belong to subgroup A of the papillomavirus family. To date, the genome of 189 HPV types have been completely sequenced. According to DNA sequence analysis, HPVs have been divided into five genera—α, β, γ, Nu, and Mu—each with different life cycle characteristics and related diseases (8–10). Epidemiological studies show that HPV types 16, 18, 31, 33, 35, 39, 45, 51, 52, 56, 58, and 59 are carcinogenic, and HPV68 are probably carcinogenic (6). Among them, HPV16 is the most prevalent worldwide and the major cause of HPV-associated cancers (11).

**Figure 2:**
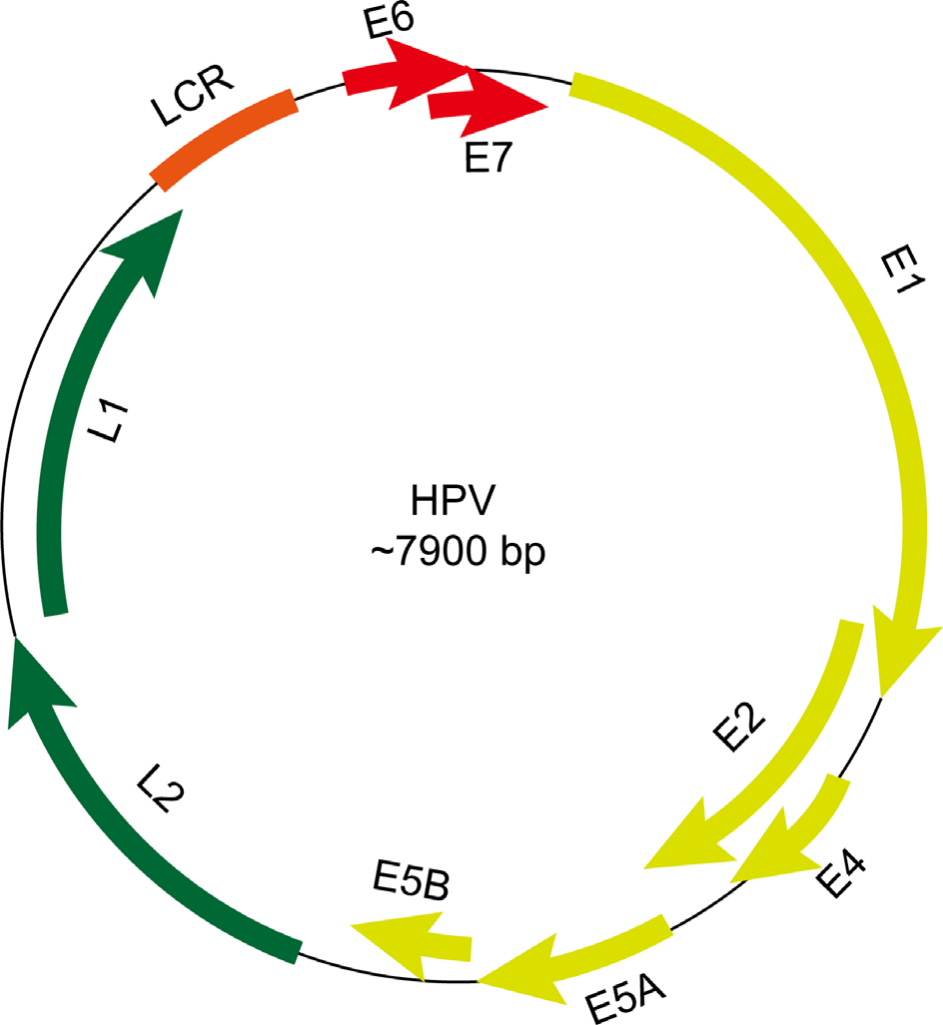
Schematic view of gene structure of human papillomavirus (HPV) genome. The HPV genome has an early transcribed region encoding six early proteins, including E1, E2, E4, E5, E6, and E7, a late transcribed region encoding two late proteins, including L1 and L2, a non-transcribed region containing the cis-elements necessary for replication and transcription and a small highly variable non-coding region located between E5 and L2. LCR, long control region.

The HPV genome has approximately 7800 to 7900 base pairs (bp) and a molecular weight of 5 × 10^6^ Daltons. It consists of 72 shell particles comprising three-dimensionally symmetrical icosahedrons with a diameter of about 55 nm. It has a lipoprotein-free membrane, a core, and a protein capsid. The HPV genome has four parts (**Figure 2**): an early transcribed region encoding six early proteins, including E1, E2, E4, E5, E6, and E7, a late transcribed region encoding two late proteins, including L1 and L2, a non-transcribed region containing the cis-elements necessary for replication and transcription, and a small highly variable non-coding region located between E5 and L2 (12, 13). The functions and characteristics of HPV-encoded proteins are shown in **Table 1**.

**Table 1:** HPV proteins and their function.

Gene location in HPV genome	Protein	Function
Early transcribed region, also known as the E region that consists of 4,500 bp	E1	E1 and E2 are involved in the completion of viral DNA replication and the life cycle, and play a key role in the virus’s initiation of replication
	E2	The full-length E2 protein functions as a transcriptional activator, which binds to the DNA of the upstream regulatory region to increase transcription in the early region, and the small E2 protein inhibits transcription in the early region
	E4	E4 is associated with viral replication mutations and is expressed late in the infection when the virus is assembled
	E5	E5 interacts with cell surface receptors such as EGF and PDGF and may stimulate the proliferation of infected cells
	E6	The combination of E6 and P53 causes the degradation of P53 protein and plays an important role in virus replication, host cell immortality and transformation
	E7	E7 binds to the Rb protein in the host cell and causes dissociation of the E2F-Rb complex that stimulates host cell transcription and plays a key role in viral replication, host cell immortality, and transcription
Late transcription region, also known as L region that consists of 2500 bp	L1	Major capsid protein, constituting the capsid of the virus, and involving in the proliferation of the virus
	L2	Minor capsid proteins, making up the capsid of the virus and involving in the proliferation of the virus

Cervical cancer, the fourth most common type of cancer in women worldwide, is one of the most preventable HPV-induced cancers. Unfortunately, cervical cancer remains a major public health problem affecting middle-aged women (14). Aside from cervical carcinomas, a substantial proportion of neoplasms of the vulva, vagina, penis, anus, and oropharynx are also highly correlated with HPV, mainly HPV16 (6, 15). A comprehensive strategy based on vaccination against HPV and screening of HPV is cost‐effective in almost all countries (16). Currently, the newest HPV vaccine is Gardasil-9 (Merck & Co), which can protect against nine different types of HPV: type 6, 11, 16, 18, 31, 33, 45, 52, and 58 (17). However, progress toward prevention is often frustrating because of the low access to vaccine and the limitations of use for HPV-positive cancer screening, especially in less developed countries (18, 19).

At present, the clinical detection method of HPV is mainly based on the polymerase chain reaction (PCR) method. This method can only be used to detect HPV DNA and HPV types and cannot be used to accurately predict HPV-positive cancers (20, 21). For instance, the serum DNA level of HPV has been shown to have a significant prognostic impact on advanced anal carcinoma before first-line chemotherapy, and HPV-circulating tumor DNA (ctDNA) becomes negative after chemotherapy completion (22). However, the HPV oncoproteins, especially E6 and E7, are more important during the HPV-induced carcinogenesis (23, 24). The integration and hypermethylation mechanisms of the HPV viral genome make the expression and lifecycle of the E5, E6, and E7 oncoproteins different from each other (25). These oncoproteins are therefore probably biomarkers for diagnosis and prognosis and may even be drug targets for therapeutics. Besides, their downstream factors can also be used for both diagnosis and therapeutics. For instance, the p16^INK4A^ protein, the immunohistochemical overexpression of which may be a useful screening test for HPV-induced cancers, is one of the important downstream factors of E7. In normal cells, p16 can be negatively transcriptionally regulated by active pRb. However, in HPV-positive cells, the pRb is inactivated by E7, thus resulting in the significant overexpression of the p16-encoded protein in HPV-positive cancer cells (26). Although p16 is a putative biomarker for HPV-transformed cervical neoplasia, it has some drawbacks, such as insufficient standardization and interpretation of the different immunoreactive stain, that make this method controversial (27). The possibility of HPV proteins as promising diagnostic and therapeutic targets is thus under reconsideration. Because of high affinity, high specificity, and biocompatibility, immunological methods based on antigens and antibodies are very useful to develop both diagnostic and therapeutic strategies for HPV-related neoplasms (28).

Over the decades, many kinds of HPV protein antibodies, including both polyclonal and monoclonal antibodies (pAbs and mAbs) against various types of HPV proteins, have been developed for both diagnosis and therapy of HPV-positive carcinomas (21, 29–31). Even other kinds of biomimetic antibodies, such as affibody, nanobody, intracellular single-chain antibodies (scFvs), as well as T-cell-based therapies, have also been developed for the diagnosis or therapeutics of HPV-associated cancers. In this review, we aim to summarize the frontiers in immunological methods that are developed based on the HPV proteins for both diagnosis and therapy.

## Immunological Methods Based on HPV Proteins for Diagnosis

**Figure 3:**
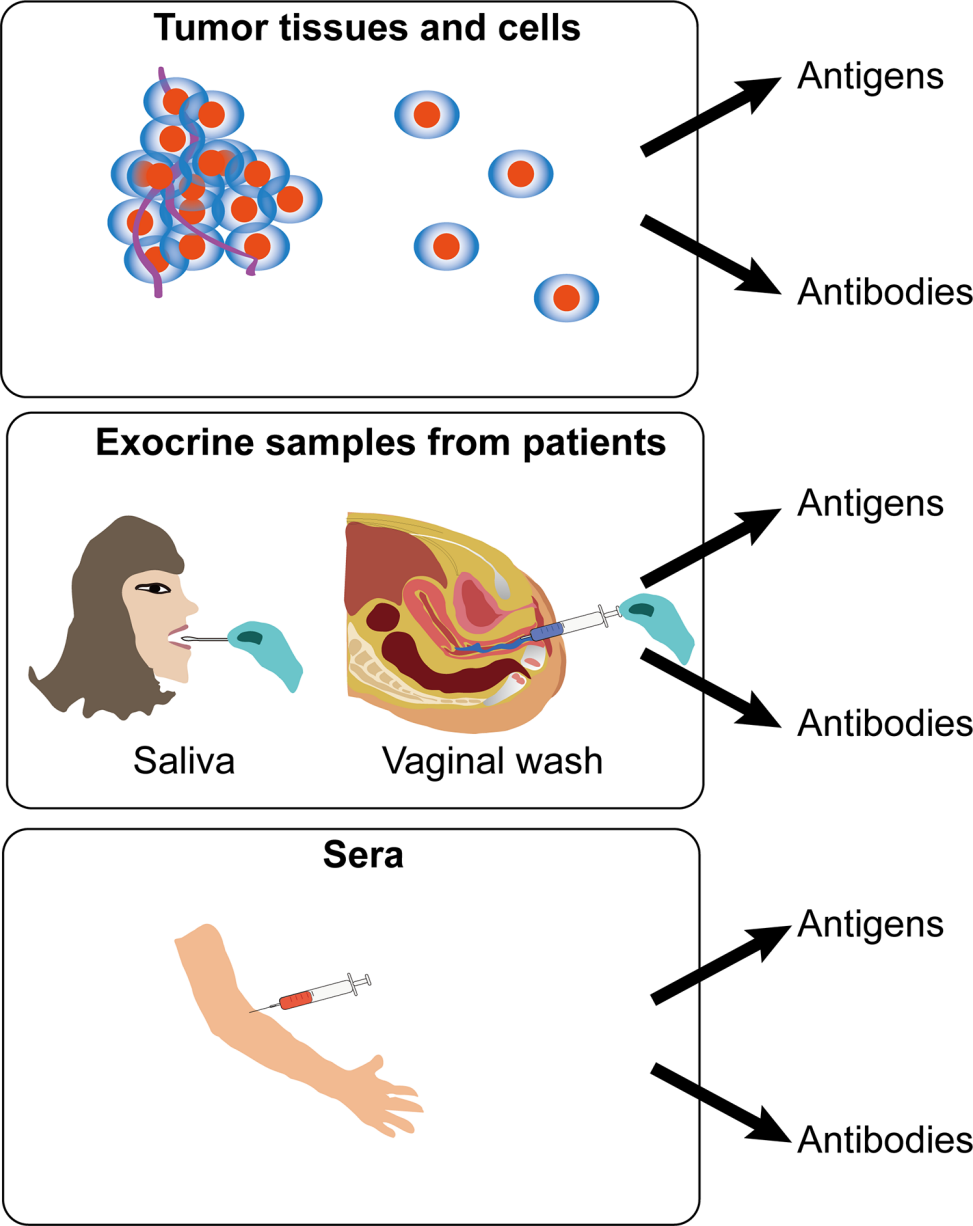
Schematic view of different samples for immunodiagnosis of human papillomavirus (HPV)-induced cancers. Detection of HPV antigen or antibodies was applied for immunodiagnosis in three kinds of sample types, including tumor tissues/cells, exocrine samples, and sera from patients.

There are many kinds of sample types including cervicovaginal, oral and serum samples for HPV detection in cervical cancers. However, a study shows that immunoglobulin-A and -G (IgG and IgA) responses to HPV16 in different kinds of sample types of women with cervical intraepithelial neoplasia (CIN) function independently of one another (32). It is therefore hard to select an appropriate sample types for the diagnosis of HPV-positive cancers. According to present studies, there are mainly three kinds of sample types, including tumor tissues/cells, exocrine samples and sera, to perform the detection of HPV antigen or antibodies (**Figure 3**).

### Detection of HPV Protein Antigens in Tumor Tissues and Cells

Tumor tissues are mainly used for validation of the detection methods based on antibodies or antigens, which can be used for prognosis, tumor typing, and medication instruction after surgery. For instance, two mouse mAbs induced by a recombinant HPV16 E7 oncoprotein were used to establish a chemiluminescent immunoassay based on a labeled streptavidin-biotin (LSAB)- enzyme-linked immunosorbent assay (ELISA) method and a luminol detection system that was suitable for the detection of HPV16 E7 oncoprotein in HPV16-positive cervical carcinoma tissues. In this method, an IgG2a-type anti-HPV16 E7 mAb was used as the detection antibody, and an IgM-type anti-HPV16 E7 mAb was used as the capture antibody (21). A Rabbit polyclonal antibody to the HPV58 E7 protein was also developed for detection of cervical cancer; it can specifically recognize the endogenous and the recombinant HPV58 E7 proteins (33).

In addition to oncogenic early proteins, many other kinds of anti-HPV antibodies, such as HPV L1 and L2, were also developed for diagnosis, evaluation of vaccines, or basal biomedical research. Two mAbs, specifically recognized as HPV18 L1 and VLPs, were used to develop an ELISA kit based on HRP-conjugated antibodies, and this method displayed good linearity, repeatability, and sensitivity for detecting HPV18 L1 pentamer and HPV18 VLP (31). Antibodies against HPV16 L1 can actively interact with the HPV18 L1, HPV31 L1, HPV45 L1, and HPV6 L1 (34). In addition to L1, L2 was also trialed for use as an antigen for diagnosis, although L2 epitopes are mainly within the canyons of pentavalent capsomers (35). However, antibody competition reveals that minor HPV L2 capsid protein residues 17–36 also locate at the surface (35). A panel of 30 mAbs, which target the N-terminus of L2 amino acids 11–200, was developed; selected mAbs were processed with enzymes, and anti-L2 Fabs were also generated (30).

The antibodies developed underpin the basis for tumor typing, diagnosis, prognosis, and further development of the therapeutic tools. However, it is assumed that detection of oncogenic antigens in tumor samples may be an auxiliary method for pathological identification and diagnosis. Besides, there are many limitations for early-stage detection or prediction of an HPV-positive tumor since the samples are acquired after the tumor size is large enough. They can be obtained only for a more accurate pathological analysis. Besides, it is difficult to acquire the valid tumor samples without an operation.

Interestingly, the exfoliated cervical cytological examination may be used for detection in the early stage of HPV-positive tumors, such as cervical cancers of the Ia stage. It can be used as an adjuvant method for cervical cytological tests. For instance, a mouse mAb panel against a C-terminal conserved sequence polypeptides of human papillomavirus (HPV) L1, a major capsid protein, by using the IHC method used for the detection of HPV in exfoliated cervical cells. The detection rate was comparable to that obtained using the commercial HPV genotyping kit currently used in clinical practice (36). Another study also showed that HPV L1 capsid detection in cervical exfoliated cells is useful for screening cervical lesions in high-risk HPV-positive women, and it may be a promising triage for high-risk women with HPV-positive atypical squamous cell of undetermined significance (ASCUS) (37). A study also used immunohistochemistry for the detection of HPV E7 protein in pre−malignant and malignant lesions of the uterine cervix, and the results showed that this method has high specificity and feasibility for detecting precancerous cells in cervical exfoliated epithelial cells. Antibody staining of cervix biopsies can indicate the pathological grade of cervical cancer and precancerous lesions (38). However, the sensitivity and specificity of HPV E6/E7 protein testing are less useful than the telomerase reverse transcriptase (hTERT) and Ki67 expression levels in distinguishing between cancerous and normal specimens (39), as this method cannot accurately detect the intraepithelial neoplasia. This leads us to look for other more accurate detection methods for the prediction of tumors after HPV infection.

Recently, biomimetic antibodies have also been designed for the detection of HPV antigens in tumors. For example, affibody molecules have a promising potential for E7 detection *in vivo*. As an antibody mimetic, affibodies contain three helical bundles with 58 amino acids in structure (40). Compared with conventional antibodies, affibodies are low in cost, highly affinitive, specifically and lowly immunogenic, small in size (around 6 kDa), and rapid in bio-distribution and elimination *in vivo* (41), making them an attractive candidate in biotechnological, pharmaceutical, and clinical developments, such as *in vivo* imaging, diagnosing, targeting, and treatment of tumors. Four HPV16 E7-binding affibody molecules (Z_HPV16 E7_127, Z_HPV16E7_301, Z_HPV16E7_384, and Z_HPV16E7_745) were screened from a phage-displayed peptide library and used for molecular imaging in tumor-bearing mice. Biosensor binding analysis showed that the four affibody molecules were bound to HPV16 E7 with very high affinity and specificity. Affibody Z_HPV16E7_384 was conjugated with Dylight755 and showed great potential to be used for *in vivo* tumor imaging and diagnosis of HPV-induced cancers (42). Further study by using whole animal imaging analysis indicated that a Z_HPV18E7_ affibody-targeted tumor tissue specifically appeared about 10 minutes after injection, and the affibody reached the highest level of tumor tissues 45 minutes after injection. At total of 24 hours after injection, the affibody still maintained a certain level in tumor tissues compared with other organs (43).

Collectively, these lines of evidence show that tumor tissues and cells are mainly used for validation of the antibodies generated. Detection of HPV protein antigens in tumor tissues and cells is not only important for tumor staging and prognosis prediction but can also be used as a promising tool for tumor imaging and orientation. However, the causality between HPV protein expression and tumorigenesis, staging and prognosis should be further interpreted. Another problem is that it is hard to detect HPV antigens for early diagnosis because the methods of obtaining samples are based on the current pathological diagnostic method used in the clinic.

### Detection of Anti-HPV Protein Antibodies in Exocrine Samples of Patients

In addition to detecting the antigens, an easier and more effective way has been developed to detect the content of anti-HPV protein antibodies. There are some different exocrine samples that have been tried for the detection of anti-HPV protein antibodies, such as saliva for OPC and vaginal wash for cervical cancer. For instance, anti-HPV E7 antibodies were detected in the saliva of patients with oropharyngeal squamous cell carcinoma and were shown to be associated with HPV status. Besides, the median longitudinal salivary E7 antibody levels decreased significantly in patients after chemotherapy treatment (44). For cervical cancer, vaginal wash can be used to detect HPV protein antibodies. An ELISA using recombinant HPV 16 E7 oncoprotein showed that levels of HPV-16 E7-specific IgG in vaginal wash were significantly higher, while the levels of the HPV-16 E7-specific IgA were lower in women undergoing radical hysterectomy for cervical cancer (HCC) or loop conization due to cervical dysplasia (LOOP), as compared with that of patients undergoing hysterectomies for other reasons (45). This may be due to the selective downregulation of local HPV-specific IgA responses in women with cervical cancer (45). This method may be used for prediction of HPV-induced cancer in the early stage in a non-invasive manner. However, since this detection method is relatively inconvenient to obtain valid samples to make sure the high detection efficacy, it is important to set up specific criteria for obtaining valid samples.

### Detection of Anti-HPV Protein Antibodies in the Sera of Patients

Anti-HPV antibodies are generated by the human body when infected with HPV and circulate in the blood, thus underpinning the basis of serological detection of HPV antibodies in the sera. However, characterization of serological anti-HPV antibody levels is challenging due to several limited factors, including complexity of oncoprotein bioactivities, pre-existing anti-HPV titers, cross-reactivity, polyclonal responses, and low affinity of non-specific antibodies. Firstly, the oncoprotein bioactivities are complex and varied. An oncoprotein can interact with many oncogenic pathways in HPV-induced cancers, but there are different levels of pre-oncogenic backgrounds in each individual. That means that the presentence of an anti-HPV antibody in sera cannot accurately predict the risk of carcinogenesis of HPV-induced cancer. Secondly, in the sera of some people who have previously been infected with HPVs but where the viruses are cleared, the serum level of the anti-HPV antibody still exists and affects the result of the prediction of tumorigenesis by using anti-HPV antibody level. Thirdly, HPV E6 and E7 have some similar sequences, and their antibodies in sera may be cross-reactive with each other and may be polyclonally responsive. Finally, the existence of non-specific antibodies in the sera can also be an obstacle for the accurate detection of anti-HPV antibodies in the sera of patients for the diagnosis of HPV-induced carcinomas.

Many efforts have been made, including exploration of the relationship between serological positivity of specific anti-HPV antibodies and carcinogenesis, the design of highly specific antigens for the successful detection of anti-HPV antibodies in the sera, and the development of a detection system with high efficacy for the quantification of HPV antibodies in the sera. From current studies, we can see there are some achievements that have been obtained.

#### Exploration of the Relationship Between Serological Positivity of Anti-HPV Protein Antibodies and Tumorigenesis

The association of carcinogenesis with serological positivity of specific anti-HPV antibodies should be explored before it is used for diagnosis. Immunological responses in different people vary, and it is difficult to set up a clear causality between serum HPV protein antibodies and tumorigenesis. However, there are many studies that have tried to find some clues.

In an ELISA assay using four HPV-16 E6–E7 peptides, the seroreactivity of patients with HPV16-associated invasive cervical cancer was greater than that of all other groups, including patients with HPV16-associated CIN and invasive cervical cancer patients without HPVs (46). High HPV E7 oncoprotein levels are necessary for cervical cancers and are apparently essential as tumor markers (47). HPV16 E7 and/or HPV18 E7 antibodies in the blood are significantly related to cervical cancer risk (48). HPV 16 E7 antibody positivity detected by using a peptide ELISA and radioimmunoprecipitation assay (RIPA) may be associated with the stage of cervical cancer (49). Previous evidence also showed that an antibody response to HPV16 E6 is more frequent than to E7, especially in cervical carcinoma at the early stages (50). However, there is also a point of view arguing that E7 functions as a valid candidate biomarker for all the stages of the malignant progression of cervical cancer (51). Antibodies to HPV16 capsids and oncoproteins E6 and E7 or types of HPV DNA in the blood samples do not appear to be useful as indicators of cervical cancer prognosis (52). However, antibodies that respond to several linear and conformational HPV epitopes are independently associated with cervical cancer, and the combined analysis of several HPV antibody responses can result in better predictive values for HPV-associated cancer (53).

In addition to exploring the causality between serological positivity of HPV proteins and cervical cancers, researchers also tried to unravel the association between serological positivity of HPV proteins and other HPV-induced cancers, such as OPC. It is found that HPV16 E1, E2, E4, E5, E6, E7, and L1-specific IgG levels in the sera are tightly associated with increased risk for HPV OPC. Among patients with OPC, HPV16 Abs are associated with tumor HPV status, particularly among HPV positive patients with no or little smoking history (54), as smoking may induce impaired antibody response in HPV16/18-infected young women below 30 years of age (55). Besides, some reports also showed that smoking and HPV infection may lead to a kind of different OPC compared with HPV-induced OPC with little or no smoking (56, 57). Positive HPV16 antibodies in the sera are strongly associated with HPV16-induced oropharyngeal cancer (58). Seropositivity of HPV16 E6 with not other HPV antigens was higher among people with than without oral HPV16 infection (59). Men with circulating L1 antibodies in the sera to HPV-6, -11, -16, or -18 are not less likely to acquire type-specific oral HPV than men without antibodies (60). Transcervical sonography and seropositivity for HPV 16 E6 antibodies are sensitive to the detection of OPC (61).

Aside from the HPV E6 and E7 oncoproteins, some other proteins may also be suitable for biomarkers of HPV-related cancers. Serological E1, NE2, and E6 antibody positivity was strongly associated with improved prognosis of patients with HPV16-positive tumors (62). HPV E2 proteins are usually expressed during the lytic stage of HPV infection in cervical cancers. Serological E2 antibody positivity was also shown to be strongly associated with tumor HPV status and prognosis (63). Sometimes, only one biomarker is unable to precisely predict the tumorignesis. However, when combined with another biomarker or other biomarkers it can be more accurate. For instance, a study showed that concurrent of antibodies against E2 and p16^INK4A^, the overexpression of which is also a biomarker of HPV-positive cervical neoplasia, was significantly associated with HPV infection and precancerous cervical lesions (64). However, some HPV proteins are quite type specific. For instance, E4 cannot be detected in HPV-18 DNA-positive CIN3 lesions but can be detected in 76% HPV16 and 55.6% HPV58 CIN3 (65).

Besides, the detection of anti-HPV antibodies before and after treatment can also be used as a prediction for the recurrence of HPV-induced tumors. The serum E7 antibody, once positive, could be detected for a long time after surgical removal of the cancers (66). A study showed that E6 and/or E7-positive/p16-positive cases have better disease-specific and recurrence-free survival rates compared with E6-/E7-/p16- cases by using a GST capture ELISA system in HPV-positive head and neck cancer (HNC) (67).

Nevertheless, E6 and E7 antibody levels undergo decay after cervical cancer treatment (68). Recurrence of squamous high-grade intraepithelial neoplasia (VIN3), which happens in approximately 30% of women after treatment, was less frequent among those with natural HPV16 antibodies in the sera (69). Antibodies against E6 and E7 (HPV16/18/31/33/35), E1 and E2 (HPV16/18) were assessed in the sera of patients with neck lymph node metastasis of squamous cell carcinoma (SCC) from unknown primary tumor (NSCCUP) and in follow-up sera from five patients. The results showed that HPV antibody levels decreased after curative treatment. Recurrence was associated with increasing levels in an individual case. However, HPV-seropositive patients had a better overall and progression-free survival (70). These results indicate that the anti-HPV antigen antibodies in the sera of patients with HPV-induced tumors may be more complex than previous expected.

A similar phenomenon is seen in HPV-positive oropharyngeal cancer (HPV-OPC). An increased level of pretreatment log-unit E6 titer was significantly associated with increased risk of disease recurrence of HPV-OPC (71). After treatment, average serum E6 and E7 antibody levels decreased significantly over time in the sera of patients with HPV-OPC (71). Patients with HPV-OPC whose disease recurs have a lower clearance of E6 and E7 antibodies than patients who do not have recurrence. The ratio of E7 antibodies at disease recurrence compared with baseline is potentially a clinically significant measurement of disease status in HPV-OPC (72). Another study showed that HPV16 E6 antibody levels decrease after treatment in patients with HPV-OPC, but most cases remain seropositive for up to 2 years. HPV16 E6 antibody levels at diagnosis did not appear to be a strong predictor of recurrence (73). This evidence makes the prognostic value of E6/E7 antibodies levels before and after treatment of HPV-driven cancer controversial (74). We suspect that the levels of the anti-HPV antigen antibodies in the sera of patients with HPV-induced tumors may be dynamic and may be due to different immune responses of different people. A more comprehensive study should therefore be performed for the better understanding of the causality between HPV antibody serological positivity and the diagnosis and prognosis of HPV-induced cancers.

High serological positivity of anti-HPV antibodies may be one cause of HPV infection, but this leads to two different results for carcinogenesis. One is that patients with higher serological positivity of anti-HPV antibodies may be caused by too much virus load in the body, resulting in high-risk of carcinogenesis. The other is that patients with higher responses of anti-HPV antibodies may be due to better patient immune systems, resulting in a quicker elimination of viruses in the body and a decreased possibility of carcinogenesis. For instance, serological positivity for IgA and IgG in the patients with cervical neoplastic lesions showed higher titers than those in the normal group (75). This complexity of anti-HPV antibodies is therefore an obstacle for the development of a serological method to detect anti-HPV antibodies for an effective diagnosis of HPV-associated neoplasms. This may be the reason why 20–40% of patients with HPV-16 DNA-positive cervical carcinoma lack serum antibodies against E6 or E7 or both since this kind of absence of anti-HPV 16 E6 and E7 antibodies in patients with HPV-induced cervical cancer is not due to the sequence variations (76). Besides, this may also be one of the possible reasons why controversial evidence is shown by using antibody levels in prediction of the recurrences of HPV-associated tumors.

#### Design of Highly Specific Antigens for the Successful Detection of Anti-HPV Protein Antibodies in the Sera

The successful detection of anti-HPV antibodies in the sera is also based on the use of highly specific antigens. HPV-16-like particles (VLPs), which are similar to native virions in structure but have no viral genome, were firstly used for reaction with the IgA and IgG responses in the sera. For example, VLPs were tested by ELISA for detecting antibodies in the sera of HPV 16 in women with cervical cancer and CIN, and the results showed that this assay using HPV-16 VLP may be useful as a diagnostic tool to supplement cervical cytological tests (77). However, since the expression modes of HPV proteins are highly different during the life cycle of HPV infection, integration and replication, different antigens also play different roles during HPV-induced carcinogenesis. The most important thing is therefore to figure out which protein is the specific biomarker for HPV-associated tumors. What is more, as the cross-reactivity and polyclonal response of anti-HPV antibody are also important for accurate detection, it is important to develop highly specific antigens for the detection of serum anti-HPV antibodies. Many more specific HPV antigens are used for developing new methods for the diagnosis of serum anti-HPV antibodies. Antibodies to HPV-16 E6 and/or E7 represent a more specific biomarker than anti-HPV-16 VLP of an HPV-related head and neck cancer (HNC) (78). L1/L2 VLPs and *in vitro*-translated E6 and E7 proteins of HPV-16 were also used and showed high serological positivity in the sera of a large amount of patients with cervical carcinoma generated (79), indicating that these HPV-16-associated proteins might be specific markers that are useful in an adjunctive diagnostic assay and a seroepidemiologic study of HPV-related cervical neoplasia. In particular, HPV-16 E7 protein seems to be valuable in the monitoring of antibody for the proper management of cervical cancers (79).

In addition to VLPs that similar with natural proteins, chimeric particles containing HPV-16 L1 protein fused with E6 and E7 seroreactive epitopes show a much better effect for detection of IgG antibodies in the sera of patients HPV16-positive CIN1 than those obtained with VLPs containing only the HPV-16 L1 protein (80). A recombinant HPV16 E7 and the N-terminal and C-terminal fragments of gp96 (NT-gp96 and CT-gp96) protein were used in the Western blot and ELISA methods to test serum antibody, and the results showed significantly higher levels of these markers in cervical cancer patients in squamous cell carcinoma only but not in adenocarcinoma and control groups (81).

Intriguingly, some biomimetic peptides are also used in serological detection of anti-HPV antibodies. Recently, a nonapeptide (16L1) derived from the HPV-16 major capsid protein was used in an ELISA system to detect potential cross-reaction of serum IgG and cervical IgA antibodies HPV-associated low-grade squamous intraepithelial lesions (LSIL) and cervical cancer patients. The results showed that the 16L1 peptide is a high-risk epitope that induces cross-reactive antibodies in patients with high-risk, but not low-risk, HPV-induced LSIL, indicating that this method is suitable for distinguish high- and low-risk infected women in the stage of low grade (82).

In summary, the selection of valid natural HPV antigens and design of biomimetic peptides are important for better detection of HPV protein antibodies. It is therefore urgent to explore how the antibodies circulating in the blood are induced by the HPV proteins, and the epitope profiles should be widely studied for a better understanding of the immunological responses upon HPV infection and what the differences in immunological responses during carcinogenesis are compared with those of the non-tumorigenic HPV infection.

#### Development of Detection System With High Efficacy for the Quantification of Anti-HPV Protein Antibodies in the Sera

The magnifying and accurate detection system used also accounted for the application of HPV antibodies detection. Currently, many detection methods for the diagnosis and prediction of HPV-associated tumors are tried in basic research. For instance, a luciferase-based detection method for determining serum antibodies is possible for the diagnosis of HPV-associated head and neck squamous cell carcinoma (83). A new LSAB capture ELISA method based on the recombinant HPV16/18 E7 oncoproteins was used to investigate anti-HPV E7 antibody prevalence in the sera of patients with cervical cancer. It is shown that this assay could potentially be used as an adjunctive tool to monitor the type of response to treatment, possibly detect antibody induction in cervical cancer patients after vaccination, and to function as a potential method to evaluate its efficacy (84). A custom HPV protein microarray based on the ELISA method, which is also called programmable protein arrays (NAPPA), was also established, displaying 98 proteins as C-terminal GST fusion proteins, representing eight antigens of two low-risk HPV types, such as HPV6, and 11 and 10 high-risk oncogenic HPV types, such as HPV16, 18, 31, 33, 35, 39, 45, 51, 52, and 58. Then, NAPPA was used to detect antigens in the sera of patients with cervical cancer and oropharyngeal cancer. The results showed this method had a great potential for rapid identification of serologic responses to 12 HPV types and could be used as a valuable high-throughput tool for measuring the breadth, specificity, and heterogeneity of the serologic response to HPV in cervical disease (85, 86).

However, the ELISA assay allows only one antigen to be evaluated at a time per well. An assay based on electrochemiluminescence (ECL) was developed by using multiplex technology from Meso Scale Diagnostics (MSD, Rockville, MD) with maltose-binding protein (MBP)-tagged E6 and E7 oncoproteins. This is a high-throughput method, but it requires lower sample quantity input with greater dynamic range to detect type-specific anti-HPV E6 and E7 oncoproteins (87).

Collectively, the present detecting methods are mainly based on chemiluminescence and ECL that can magnify the signals of immunological reactions. The success of novel detection systems also depends on the clear understanding of the biomarkers in HPV-induced cancers. The current biggest obstacle of immunodiagnosis of HPV-induced cancers is therefore the appropriate specific biomarkers.

## Therapeutics

**Figure 4:**
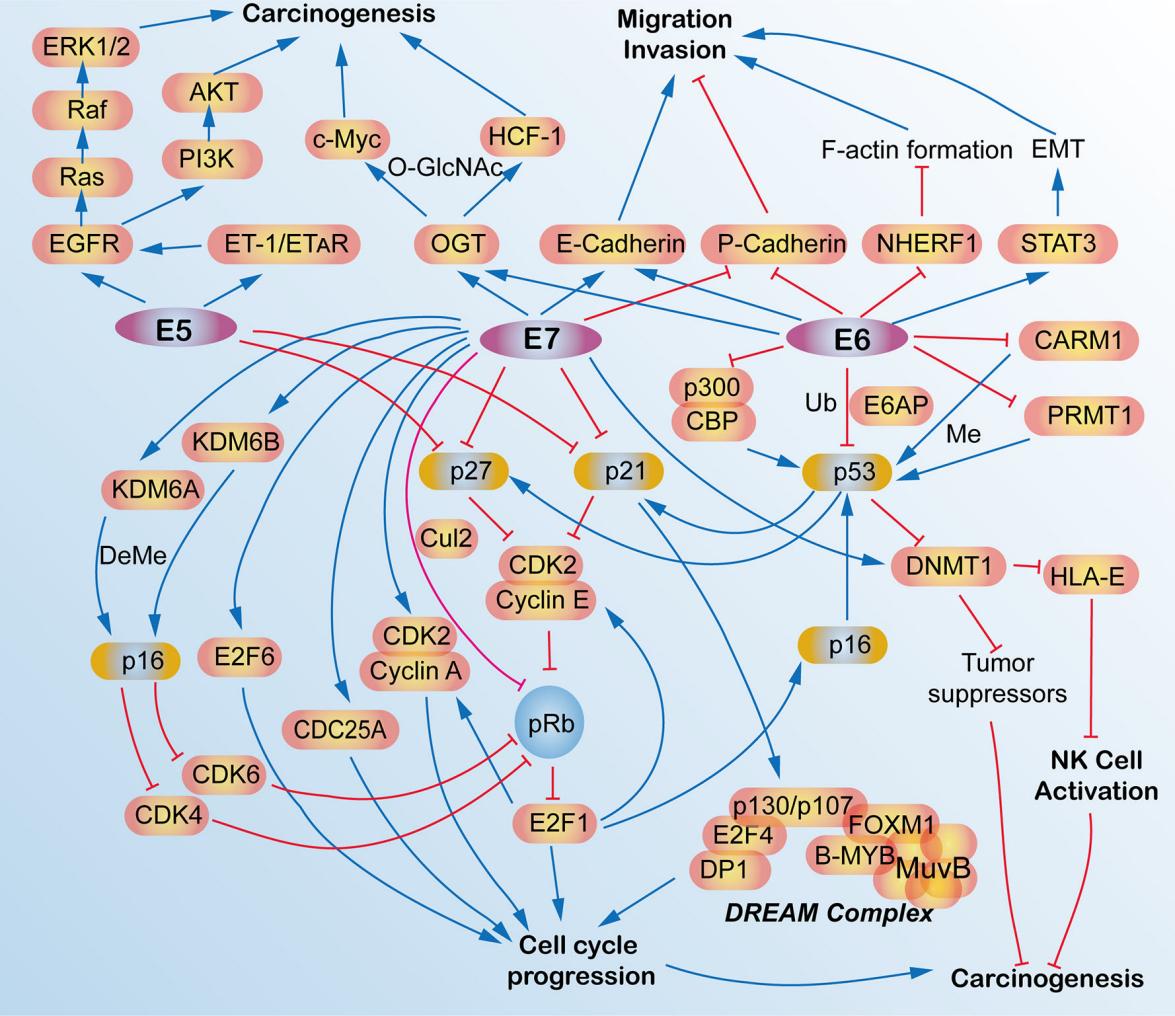
Schematic view of the molecular network and functions of human papillomavirus (HPV) E5, E6, and E7 oncoproteins in HPV-induced cancers. E5, E6, and E7 oncoproteins are responsible for the carcinogenesis and tumorigenesis of HPV-induced cancers *via* activating multiple signaling pathways, such as cell cycle signaling, EGFR pathway, migration, and invasion pathways, p53 pathway and some epigenetic pathway. AKT, AKT serine/threonine kinases; B-MYB, MYB proto-oncogene like 2, MYBL2; CARM1, coactivator-associated arginine methyltransferase 1; CBP, CREB binding protein; CDC25A, cell division cycle 25A; CDK2, cyclin-dependent kinase 2; CDK4, cyclin-dependent kinase 4; CDK6, cyclin-dependent kinase 6; C-Myc, avian myelocytomatosis viral oncogene homolog; Cul2, cullin 2; DeMe, demethylation; DNMT1, DNA (cytosine-5-)-methyltransferase 1; DP1, transcription factor DP-1, TFDP1; E2F1, E2F transcription factor 1; DREAM, dimerization partner, RB-like, E2F and multi-vulval class B; E2F4, E2F transcription factor 4; E2F6, E2F transcription factor 6; E6AP, E6-associated protein, ubiquitin protein ligase E3A, UBE3A; E-Cadherin, epithelial cadherin; EGFR, epidermal growth factor receptor; EMT, epithelial mesenchymal transition; ERK1/2, mitogen-activated protein kinase 1/2; ET-1, endothelin 1; ETAR, endothelin A receptor; F-actin, filamentous actin; FOXM1, forkhead box M1; HCF-1, host cell factor 1; HLA-E, HLA class I histocompatibility antigen, alpha chain E; KDM6A, lysine demethylase 6A; KDM6B, lysine demethylase 6B; LIN54, protein lin-54 homolog; Me, methylation; MuvB, MuvB complex of five proteins including LIN9, LIN37, LIN52, RBBP4 and LIN54; NHERF-1, Na+/H+ exchanger regulatory factor; NK, natural killer; O-GlcNAc, O-Linked β-N-acetylglucosamine; OGT, protein O-GlcNAc transferase; p16, p16^INK4A^, p14^ARF^, cyclin dependent kinase inhibitor 2A, CDKN2A; p21, p21^WAF1/CIP1^, cyclin-dependent kinase inhibitor 1A, CDKN1A; p27, p27^KIP1^, cyclin dependent kinase inhibitor 1B, CDKN1B; p53, tumor protein p53, TP53; p300, E1A binding protein p300; P-Cadherin, placental cadherin; PI3K, phosphoinositide 3-kinase; pRb, phosphorylated retinoblastoma transcriptional corepressor 1; PRMT1, protein arginine N-methyltransferase 1; Raf, Raf proto-oncogene, serine/threonine kinase; Ras, RAS proto-oncogene, GTPase; STAT3, signal transducers and activators of transcription 3; Ub, ubiquitination.

HPV oncoproteins, such as E5, E6 and E7, are not only the etiological factors for carcinogenesis for HPV-associated cancers, but some of them are constantly expressed during tumor development and exhibit malignant characteristics *via* interacting with multiple signaling pathways and regulatory modes that participate in the modulations of intraepithelial neoplasia (IN), tumorigenesis, cell cycle progression, cell survival, and metastasis (88–90) (**Figure 4**).

For instance, HPV16 E6/E7 could promote the invasive potential of cancer cells and epithelial-mesenchymal transition (EMT) *via* turning on the cadherin switch, downregulating NHERF1, and/or activating the STAT3 signaling pathway (91–93). HPV E6 protein expression enriches the differentiation (CD) 55-positive population, which are possible cancer stem-like cells that contribute to tumourigenesis and radioresistance in cervical cancer cells (94). HPV16 E6/E7 induces O-linked GlcNAcylation (O-GlcNAc) and O-GlcNAc transferase (OGT), thereby elevating c-MYC and transcriptional co-regulator host cell factor 1 (HCF-1) *via* increased protein stability, which results in tumor transformation and tumorigenesis (95, 96). E6 induces proteasome-dependent p53 degradation *via* recruiting the intracellular ubiquitin ligase E6AP (97). Recently, HPV16/18 E5 is also expressed in the early stage of carcinogenesis (98) and is shown to promote cervical cancer cell proliferation, migration, and invasion *in vitro*, and it accelerates tumor growth in animal models (99).

These oncoproteins are potential drug targets for the therapeutics of HPV-associated cancer. Among them, immunotherapy-based on HPV is very important. From current studies, we can see there are many kinds of immunotherapeutical methods or immunotherapy-like methods based on HPV studied in basal research, including anti-HPV protein mAbs, radioimmunotherapy, affitoxins, single-chain antibodies (scFvs), nanobodies, therapeutic vaccines, and T-cell-based therapies.

### Native Anti-HPV Protein mAbs for the Therapeutics of HPV-Induced Cancers

Anti-HPV E6 and HPV E7 mAbs *via* intraperitoneal or intratumoral injections in mice models significantly inhibited cervical tumor growth (100). Another study also showed that mAbs specifically targeting HPV6 E7 49-57 peptides lead to a significant tumor-suppressing effect in animal models (101). Other mAbs that target HPV E6/E7 also have an anti-tumor effect to some extent according to several reports (102–105). Theoretically, the use of anti-HPV mAbs has limited potential in cancer therapy because its size is too big to enter into the intracellular regions where the HPV oncoproteins are located. However, these samples showed that native anti-HPV mAbs also have some anti-tumor effects, and this may be because cellular turnover occurs as cervical cancer solid tumors grow, making it possible for anti-HPV E6 and E7 to be accessible to these two oncogenes *via* necrosis (100). In contrast to this, some other researchers have pointed out that the deposition of C3 complement and lymphocytes infiltration, which potentially exert significant antitumor effects, is induced by mAbs treatment rather than necrosis (105).

### Radioimmunotherapy for the Therapeutics of HPV-Induced Cancers

**Figure 5:**
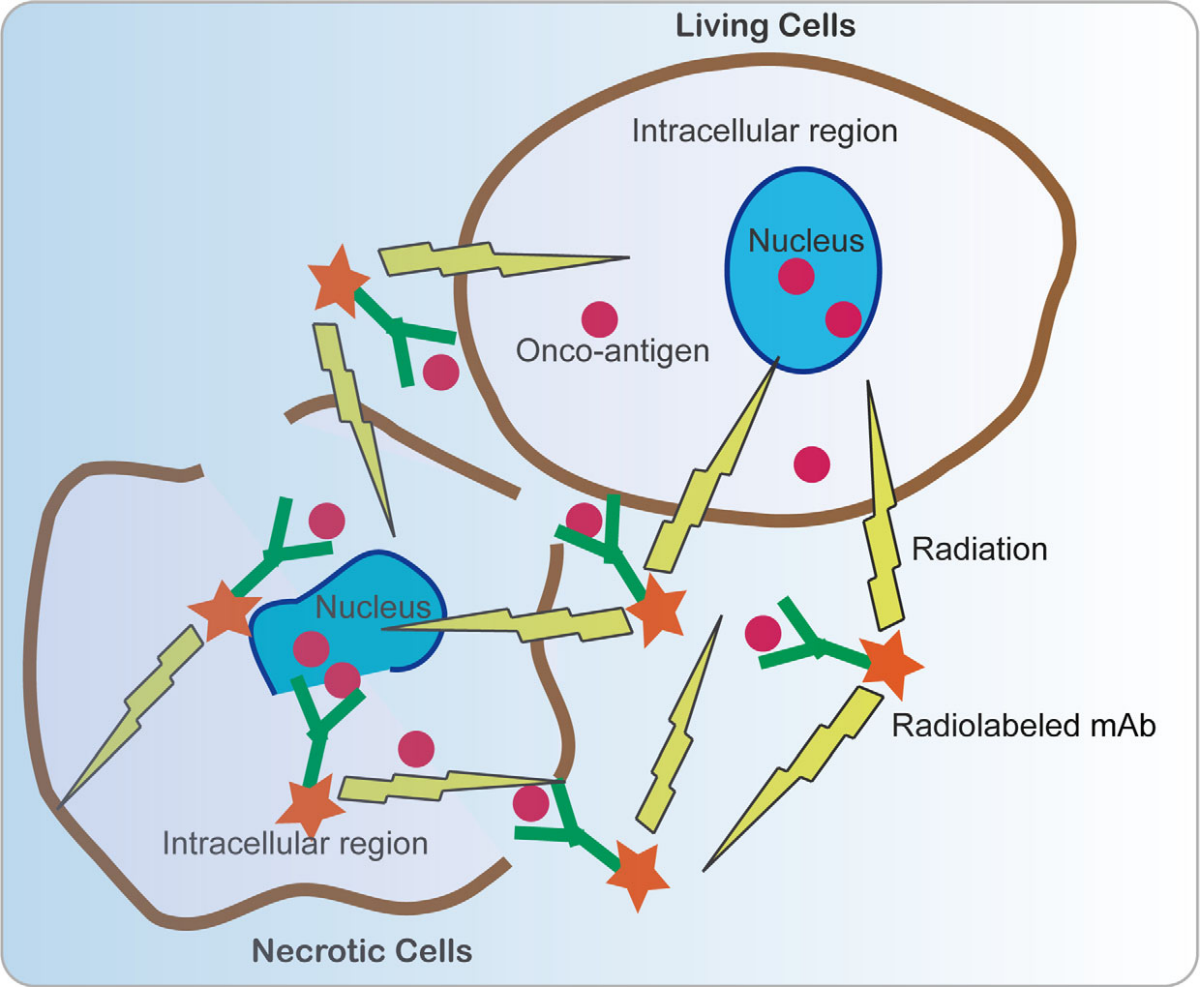
Schematic view of anti-cancer mechanism of radiolabeled mAbs. Since many non-viable and necrotic cells with permeable membranes are present in tumors, allowing mAbs to access to interact with the intracellular antigens. In addition, intracellular antigens E6 and E7 can be released from cancer cells *via* necrosis and cell turnover. E6 or E7 specific mAbs bind to extracellular E6 and E7 and deliver cytotoxic radiation to this area. Surviving tumor cells including weak or no E6 or E7 expression were killed by radiation by the “cross-fire” effect produced by radiation in 360° spheres.

Radioimmunotherapy (RIT) is a kind of therapeutic method that systemically administers radiolabeled mAbs to bind to specific tumor-associated antigens (40, 106). Conventionally, traditional RIT aims for cell-surface-associated tumor markers; however, targeting viral antigens within the tumors is fundamentally different from that because the oncotargets are of viral origin while not “self” human antigens, which minimizes cross-reactivity with host tissues. Nevertheless, the difficult condition is that the viral oncoproteins normally reside in intracellular compartments, which is thought to be beyond the reach of immunogloblulins. For instance, the E6 and E7 oncoproteins are usually located in the intranuclear location. Intriguingly, this approach really works due to many non-viable and necrotic cells with permeable membranes in tumors that allow mAbs access to interact with the intracellular antigens. In addition, due to the renewal of cells in a rapidly growing tumor, the cellular membrane has provided some crevasses for some intracellular antigens E6 and E7 to be released from cancer cells. Radiolabeled E6 or E7 specific mAbs bind to extracellular E6 and E7 and deliver cytotoxic radiation to this area. Surviving tumor cells, including weak or no E6 or E7 expression, were killed by radiation *via* the “cross-fire” effect produced by radiation in 360° spheres (**Figure 5**) (107).

For example, E6 and E7 oncoproteins in experimental cervical cancer can be targeted by using radiolabeled mAbs with a beta-emitter 188-Rhenium (^188^Re) attached to HPV E6, making them selective mediators of tumor destruction even in experimental cervical tumors expressing low levels of E6 (102, 103). In addition, the radiolabeled anti-HPV E6 mAb alone and in combination with MG-132, a proteasome inhibitor, was used to treat cervical tumor-bearing mice and showed a significant suppression of tumor growth (103, 104). Radioimmunotherapy based on ^188^Re directed toward HPV E7 oncoprotein also inhibits experimental tumors growth (105). Besides, beta emitters lutetium-177 (^177^Re) are equally effective for radioimmunotherapy based on an anti-HPV E6 mAb in HPV-positive cervical cancer (108). These results mean that radiotherapies based on both E6 and E7 are probably potential therapeutic methods for HPV-positive cancers.

### Affitoxins for the Therapeutics of HPV-Induced Cancers

In addition to applications in tumor imaging and diagnosis, affibodies can also be used as therapeutic tools for tumor treatment. Clinical and pre-clinical studies showed that several affibodies have been developed to target some oncogenic proteins, such as human epidermal growth factor receptor 2 (HER2), epidermal growth factor receptor (EGFR), and insulin-like growth factor type 1 (IGF1R) (109, 110). These affibodies showed a promising future for early-stage cancer diagnosis and treatment in the clinic. Interestingly, affibodies were also applied in HPV-positive tumors *via* targeting HPV oncoproteins. In a mouse cervical cancer model, Z_HPV18E7_ and Z_HPV16E7_ affibodies connected with *Pseudomonas* exotoxin, also known as affitoxins, were able to deliver *Pseudomonas* exotoxin, a clinically used anti-cancer agent to tumor tissues effectively, showing great potential for HPV-induced cancer treatment (43, 111). These results exemplify the potential use of affitoxins for HPV-induced cancers.

### Intracellular Single-Chain Antibodies (scFvs) for the Therapeutics of HPV-Induced Cancers

**Figure 6:**
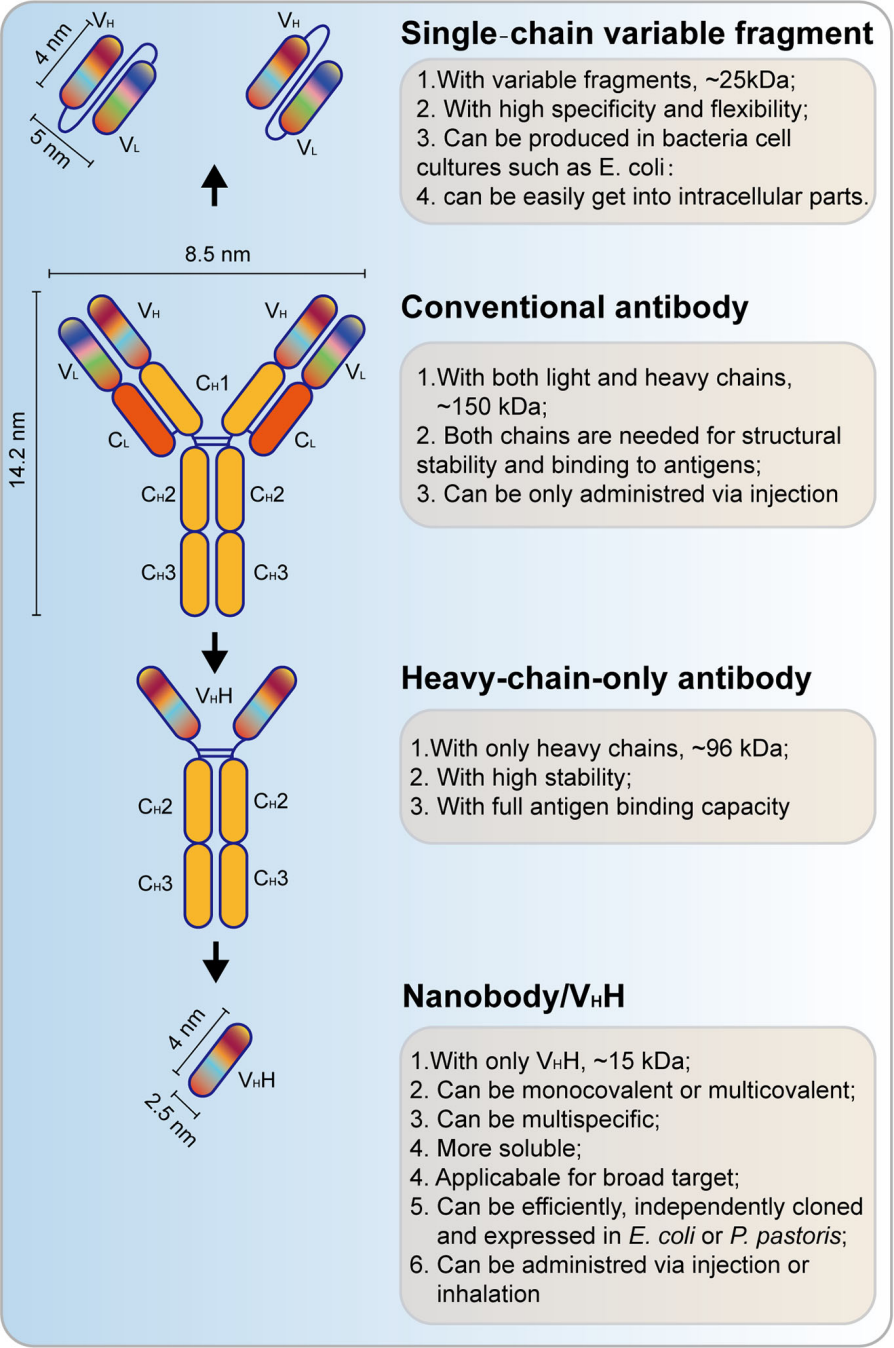
Schematic view of single‐chain variable fragment (scFv) antibody and nanobody. V_H_, heavy chain variable domain; V_L_, light chain variable domain; C_H_, heavy chain constant domain; C_L_, light chain constant domain; V_H_H, single-domain antibody.

Single‐chain variable fragment (scFv) antibodies are the smallest immunoglobulins, but they have a high antigen‐binding affinity (**Figure 6**). Compared with mAbs, scFvs can access the intracellular region and bind to intracellular oncoantigens. It can be well produced by using prokaryotic expression in *Escherichia coli*. Applications of scFv against fibroblast growth factor receptor (FGFR) and fibroblast growth factor 1 (FGF-1) have shown a great anti-tumor effect both *in vitro* and *in vivo* (112–114). Recently, scFvs have also been applied in HPV-induced neoplasms by targeting oncoproteins, including HPV E5, E6, and E7. The HPV16 E5 protein is a small hydrophobic protein, the expression of which generally decreases as the infection progresses to malignancy. ScFvs were used to recognize HPV16 E5 in W12 cells by fluorescent microscopy and its colocalization with one of its host substrates (115). Functional scFvs against the E6 oncoprotein can also be produced in *E. coli*, and it impairs growth of HPV16-positive tumor cells in mouse models (116, 117). Anti-HPV16 E7 scFvs expressed in HPV DNA-containing cell lines showed a significant decrease in cell proliferation *via* altering the level of HPV16 E7 oncoprotein (118). Besides, anti-HPV16 E7 scFvs also exerted an *in vivo* antitumor effect (119). Two scFvs binding E7, designed and generated by using distinct but overlapping epitopes, were selectively expressed in the nucleus and the endoplasmic reticulum (ER) of cervical cancer cells, leading to the selective inhibition of tumor growth of these cells. These two scFvs do not correspond to the pRb binding site, but they can be non-competitively inhibited by pRb (120). These results showed a great potential for scFvs in the treatment of HPV-positive tumors.

### Nanobodies for the Therapeutics of HPV-Induced Cancers

Different from scFvs, heavy chain antibodies (HCAbs) are another kind of small antibodies (approximately 15 kD) that are naturally devoid of light chains, the antigen-binding fragment of which consists of only one single domain referred to as nanobody (Nb) or the heavy chain antibody variable region (V_H_H, **Figure 6**) (121). This kind of antibody, which is also referred to as camelid single-domain antibody (sdAb), is inspired by the HCAbs that can be found in the *Camelidae* family (122). Nb is more soluble and stable than conventional antibodies, and a high yield can be found in bacteria (*E. coli*) or yeast (*Pichia pastoris*). Recently, Nbs also function important therapeutic approaches for cancer treatment (123). Nbs that target HPV L1 have been successfully achieved (124). Importantly, Nbs are also applied for targeting of HPV E7 in cervical cancers. A phage-display approach was used to select the high-affinity HPV16 E7-specific Nbs and expressed by using prokaryotic expression. Then, a high-affinity Nb was expressed in cervical cancer, and it induced a significant decrease of tumor growth (125). These studies underpin the basis of the applications of Nbs in HPV-induced tumors.

### Therapeutic Vaccination for the Therapeutics of HPV-Induced Cancers

Unlike preventive vaccines, therapeutic vaccines fight against an existing disease rather than immunizing for protection against future disease by utilizing a patient’s own immune system. These therapeutic vaccines deliver antigens to antigen-presenting cells (APCs), where these antigens are processed and digested into short peptides by proteasome, which can stimulate antigen presentation *via* major histocompatibility complex class I and II (MHC I/II), leading to CD8+ cytotoxic T-cell or CD4+ helper T-cell responses. This method has also been trialed for cancer therapy (126). Recently, this method has also been introduced for the therapy of HPV-induced tumors (127). There are several kinds of therapeutic vaccines, including live vector (bacterial or viral vector), protein or peptide, nucleic acid, and whole cell-based vaccines (128).

#### Live Vector-Based Therapeutic Vaccines

Although live vector-based vaccines may pose a safety risk, they can induce strong cellular and humoral immune responses. The vectors can be either bacteria, such as *Listeria monocytogenes, Lactobacillus lactis, Lactobacillus plantarum*, and *Lactobacillus casei*, or viruses, such as adenoviruses, adeno-associated viruses, alphaviruses, and vaccinia viruses. They can infect macrophages and stimulate immune responses that kill tumor cells. *Listeria*-based E7 vaccines were reported as a promising method for the treatment of HPV E6/E7-expressing autochthonous solid tumors in mice (129). Besides, *Listeria* can secrete a kind of toxin, listeriolysin O (LLO), which can make the bacteria escape from phagosomal lysis and decrease the CD4+FoxP3- and CD8+ regulatory T cells, resulting in HPV vaccine-mediated tumor regression (130). A phase I study on the intravenous *Listeria monocytogenes-*based fusion vaccine consisting of HPV16 E7 antigen and LLO in cervical cancer showed a reduction of total tumor size that was observed in four of 15 patients, but this method also induced some adverse effects, such as pyrexia, vomiting, chills, headache and anemia, nausea and tachycardia, and musculoskeletal pain (131, 132). Further study showed that oral administration of the *Listeria casei*-based HPV16 E7 vaccines exerted a remarkable E7-specific cell-mediated immune responses in patients with HPV16+ CIN III lesions (133).

In addition to bacterial vectors, viral vector-mediated vaccines against HPV oncoantigens are also tested for cancer therapy. Decades ago, a live recombinant vaccinia virus vaccine encoding HPV16 and 18 E6 and E7 oncoproteins, also known as TA-HPV, induced a specific cytotoxic T-cell response in three patients and eliminated tumors of two patients at 15 and 21 months after vaccination (134). Furthermore, TA-HPV was shown to reduce HPV-associated lesion size and to stimulate HPV 16 E6 and E7-specific T-cell immunity in patients with HPV-16+ vulval intraepithelial neoplasia (VIN) grade III and a single patient with HPV-16+ vaginal intraepithelial neoplasia (VAIN) grade  (135). In addition to vaccinia virus, a replication-deficient adenovirus-encoding fusion vaccine consisting of calreticulin (CRT) and HPV16 E7 can also eliminate E7-expressing tumors in mice (136). Other kinds of chimeric vaccines mediated by viruses, such as adenovirus vaccine encoding hepatitis B virus surface antigen (HBsAg) and HPV16 E7 proteins as well as alphavirus vaccine encoding recombinant Semliki Forest virus (rSFV) particles and HPV16 E6/E7, can increase anti-HPV E7 antibody and cytotoxic T-cell responses in mice (137–139). Importantly, a clinical study showed that a modified vaccinia virus Ankara (MVA)-based vaccine, which has a suspension of MVATG8042 particles with attenuated recombinant MVA that carry sequences encoding modified HPV-16 E6/E7 and human IL-2, also referred to as TG4001, can clear both HPV16 DNA and mRNA, with no recurrence of high-grade lesions one year after treatment in patients with CIN 2/3 (140). In addition to HPV E6 and E7, HPV E2 was also used in an MVA-based vaccine and the results demonstrated a strong therapeutic effect *via* stimulating the immune system in a phase III clinical study for the treatment of HPV-induced anogenital intraepithelial lesions (141).

#### Peptide/Protein-Based Therapeutic Vaccines

Another important kind of vaccine is peptide/protein-based vaccine. Those antigens derived from HPV can be processed by dendritic cells and presented on MHC class I/II molecules, which further can stimulate CD8+ or CD4+ T cell responses (142). They are safe, stable, and easy to produce, but they have poor immunogenicity. A clinical study showed that HPV16-synthetic long-peptide (SLP) vaccination combined with standard carboplatin and paclitaxel (CarboTaxol) chemotherapy can significantly improve immunity *via* fostering robust T-cell responses (143). Other several HPV16 peptide-based vaccines, such as TA-CIN (a subunit vaccine composed of HPV16 E6E7L2 fusion protein) (144), PepCan (a therapeutic HPV vaccine containing four synthetic peptides covering HPV-16 E6 and Candin) (145) and GTL001 (a therapeutic HPV protein vaccine targeting both HPV16 and HPV18) (146) showed remarkable potentialities for HPV-positive malignancies. Recently, a well-documented HPV-E7 SLP) therapeutic vaccine was performed to conjugate to ultra-small polymeric nanoparticles (NP), thus enhancing their antitumor efficacy in different HPV-positive tumor-bearing animal models. This synergetic effect is due to a larger pool of E7-specific CD8+ T cells with increased anti-tumor efficacy induced by conjugated E7 SLPs than unconjugated ones. A robust infiltration of CD8+ T cells was also observed at the tumor site treated with conjugated E7 SLPs; however, concomitant accumulation of regulatory T cells (Tregs) did not occur, leading to a higher CD8+ T-cell to Treg ratio. The NP-E7 SLPs thus exemplify a “solid-phase” antigen delivery strategy that can be more effective to treat viruses-related tumors than a conventional free-peptide (“liquid”) vaccine (147). These results highlight the potential of using therapeutic vaccination against solid tumors like HPV-induced cancers.

#### Nucleic Acid-Based Therapeutic Vaccines

Nucleic acid-based vaccines are also easy to be produced. Recently, two HPV E5-based versions of DNA vaccines carrying a whole E5 gene or a synthetic multiepitope gene were improved by fusion to sequence of potato virus X (PVX) coat protein. This vaccine candidate was then challenged in a new luminescent animal model based on a C3-Luc cell line, and a strong cellular immunity was induced, resulting in eliciting strong anti-tumor effects (98). Unlike DNA vaccines, which are stable, the RNA vaccines are not stable. However, combining RNA replicons and DNA vaccine into a DNA-launched RNA replicon, or ‘suicidal DNA’, can overcome this. Unfortunately, they lead to poor immunogenicity. The incorporation of genes encoding anti-apoptotic proteins and the use of a flavivirus Kunjin (KUN) vector are therefore designed to deliver the replicons (142).

#### Whole Cell-Based Therapeutic Vaccines

Dendritic cell- and tumor cell-based vaccines are two main kinds of whole cell-based vaccines. DC-based HPV vaccines load the DCs with HPV antigens *ex vivo* and subsequent deliver DCs to the patient (148). A phase I clinical study showed that a DC-based vaccine can promote HPV-specific humoral response in patients with stage Ib or IIa cervical cancer after vaccination, and it was safe and well tolerated by patients (149). Tumor cell immunogenicity *in vivo* has been improved through the increased expression of immune-modulatory proteins, such as cytokine genes IL-2, IL-12, and granulocyte macrophage colony stimulating factor (GMCSF) (150, 151). But it is not suitable for HPV-induced tumors because it can increase the risk of new tumor formation. Besides, tumor-cell-based vaccines may not be based on HPV antigens since their targets are not clear.

### T-Cell-Based Therapies Against HPV Onco-antigens for the Therapeutics of HPV-Induced Cancers

Over the last decades, tumor immunology has expanded our understanding on the relationships between the tumor cells and tumor environment where immune cells, such as cytotoxic T cells, helper T cells, and regulatory T cells, are located. These cells are final effectors of immune response that can induce tumor regression. Recently, there are several types of T-cell based therapeutics also known as T-cell-based therapeutic vaccines or adoptive T-cell therapies (ACTs), such as tumor-infiltrating cells (TILs), engineered T-cell receptor (TCR) T cells, and chimeric antigen receptor T (CAR-T) cells, that has been tested in experimental research and clinical trials (128).

#### Tumor-Infiltrating Cells (TILs)-Based Therapies

TILs are T cells were isolated from tumors, expanded *ex vivo*, and then infused back to patients to regress the tumor development. This method has been used in the treatment of melanoma, gastrointestinal cancer, and lung cancer (152). Importantly, it has also been tested in epithelial tumors such as HPV-induced cervical cancer. TILs selected for HPV E6 and E7 oncoantigens induced remarkable tumor regression in patients with metastatic cervical cancer (153). A single-center phase II study further made sure that HPV-TILs can regress the progression of HPV-induced tumors, including cervical cancer, oropharyngeal cancer, and anal cancer (154). Besides, PD-1–expressing TILs was recently reported as a favorable prognostic and therapeutic biomarker in HPV-associated head and neck cancer (155). CD8+ and FOXP3+ TILs were also shown to be correlated with clinical outcome and HPV status of tonsillar cancer (156). These results imply that TILs targeting HPV E6/7 and other factors, such as PD-1, CD8, and FOXP3, may be more effective.

#### Engineered T-Cell Receptor (TCR) T Cells-Based Therapies

In addition to TILs selected from tumors, engineered TCR T cells may be more powerful and easier to obtain. Engineered TCR T cells are peripheral blood T cells genetically engineered to express a tumor-targeting surface or intracellular/nuclear proteins processed and presented on the cell surface *via* MHC molecules and to help overcome immune tolerance. Recently, several phase I/II studies showed that TCR gene therapy would be the most promising immunotherapeutic method for the treatment of HPV-positive cancers. T-cell receptor gene therapy targeting human papillomavirus-16 E6 or E7 can induce a remarkable regression of HPV-positive epithelial cancers including cervical, oropharyngeal, anal, vulvar, vaginal, and penile cancers, in two phase I clinical trials (157, 158). The most recent study (159, 160) showed that 12 patients with metastatic HPV16-positive epithelial cancer (six had cervical cancer, four had anal cancer, one had oropharyngeal cancer, and one had vaginal cancer) who had received prior platinum-based therapy, received autologous genetically engineered T cells expressing a T-cell receptor directly targeting HPV16 E6 (E6 T-cell receptor T cells). After 1 month of treatment, all patients demonstrated high peripheral blood engraftment with E6 TCR T cells. Two of 12 patients (16.7%) had objective tumor responses, one of whom (female patient with three lung metastases) had one tumor that was completely regressed; two were partially regressed and were subsequently resected. There were no dose-limiting toxicities observed and no maximum tolerated dose established in this study. These results show that engineered TCR T cells-based therapies that target HPV oncoantigens have great potential for HPV-induced neoplasms.

#### Chimetric Antigen Receptor T (CAR-T) Cells-Based Therapies

There is also another T-cell based therapy called CAR-T cells. A chimeric antigen receptor is designed and expressed in the T cells to provide an appropriate co-stimulatory signaling that can efficiently active effector T cells. This method has been widely tested in hematological malignancies and achieved promising effects. It has been approved by the U.S. Food and Drug Administration (FDA) for the treatment of several types of hematological malignancies. Although this method is less successful in solid malignancies, a phase I/II clinical study (NCT03356795) is performing for intervention of CAR-T against cervical cancer positive to GD2, PSMA, Muc1, Mesothelin or other markers. If this method is efficient, then CAR-T against HPV oncoantigens may also be promising therapeutics for HPV-positive cancers.

## Conclusions and Perspectives

From above, we can conclude that immunodiagnosis and immunotherapeutics has been rapidly developed for HPV-induced tumors. The immunodiagnostic methods are exploited to detect the antigen in tumors or cells *in situ* or detect the antibody in the internal body fluid, such as sera, or external body fluid, such as saliva and vaginal wash. The immunotherapeutical methods including mAbs, radiolabeled mAbs, affibodies, nanobodies, therapeutic vaccines, and T-cell based therapies are developed to target the HPV oncoproteins, including HPV E5, E6, and E7. For the immunodiagnosis, detecting anti-HPV protein antibodies may be of promise because this method is relatively easier and more accurate. However, the method should be combined with other diagnostic methods, such as HPV typing, based on HPV, DNA, or other molecular targets, such as p16, which may be more accurate. For the immunotherapeutics, the T cell-based and novel antibodies-based therapies may be a promising method for the therapeutics. However, because of the heterogeneity of the HPV-induced cancers, it is very difficult to find a universal way to cure all HPV-induced cancers. It is therefore urgent to perform a more comprehensive study to develop a better molecular typing method that is based on HPV antigens. Although it is not easy, it will hold a great promise for the use of immunodiagnosis and immunotherapeutics based on HPV for HPV-induced cancers.

The accuracy of immunodiagnostic and immunotherapeutic methods is based on the specific antigen and antibody that are specifically associated with the pathological progression of HPV-associated tumors. From current studies, HPV E6 and E7 are the two most important protein targets for both diagnosis and therapy. However, the biggest problem is that the specificity of detecting these proteins and their corresponding antibodies need to be further optimized. More basal research should thus be performed to explore the profiles of the expression, functions, and regulations of these HPV oncoproteins in HPV-induced oncogenesis. Afterwards, more clinical trials should be done to validate the results obtained from the basal research. It is believed that immunodiagnosis and immunotherapeutics based on HPV will hold great promise for the prevention of people with HPV infection and therapy for patients with HPV-associated tumors.

## Author Contributions

ZD and RH reviewed the literature, designed the tables and figures, and wrote the manuscript. YD, LT, JD, LL, LB, and YM collated the literature and proofread the manuscript. HC scrutinized and revised the text. All authors contributed to the article and approved the submitted version.

## Funding

We are grateful for funding support from the National Natural Science Foundation of China (Nos. 81902664, 81872071, and 81672502), the Fundamental Research Funds for the Central Universities (Nos. SWU120009 and XYDS201912), the National Key Research and Development Program of China (Nos. 2016YFC1302204 and 2017YFC1308600), the Experimental Technology Development Fund Project of Chongqing University of Technology (No. sk201812), the Opening Project Funding from the State Key Laboratory of Silkworm Genome Biology (No. sklsgb161718-5) and the Natural Science Foundation of Chongqing (No. cstc2019jcyj-zdxmX0033).

## Conflict of Interest

The authors declare that the research was conducted in the absence of any commercial or financial relationships that could be construed as a potential conflict of interest.
